# Supplementary material for: Physical Inactivity and Sedentarism during and after Admission with Community-Acquired Pneumonia and the Risk of Readmission and Mortality: A Prospective Cohort Study
Source: J Clin Med. 2022 Oct 7;11(19):5923. doi: 10.3390/jcm11195923 (PMC9571502; doi:10.3390/jcm11195923)
Supplement: Supplementary file 1 [file jcm-11-05923-s001.zip › Supplementary - major revision.pdf]

Supplementary

Table S1. Physical activity level prior to admission between patients included before the COVID-19 lockdown compared to patients included during or after the lockdown.

	Before COVID-19 lockdown (n = 55)	During or after COVID-19 lockdown (n = 96)	P-value
Low, n (%)	42 (76.4)	79 (82.3)	0.380
Moderate, n (%)	10 (18.2)	10 (10.4)	0.176
High, n (%)	3 (5.5)	7 (7.3)	0.662

Comparisons were made with Chi-squared tests.

Table S2. Cause of 30-day readmission

Cause of readmission	
Pulmonary, n (%)	19 (51.4)
Cardiovascular, n (%)	6 (16.2)
Malignant, n (%)	3 (8.1)
Infection, n (%)	3 (8.1)
Neurological, n (%)	2 (5.4)
Other, n (%)	4 (10.8)
